# Intersubunit communication in glycogen phosphorylase influences substrate recognition at the catalytic sites

**DOI:** 10.1007/s00726-023-03362-6

**Published:** 2024-02-10

**Authors:** Nahori Kamada, Ayato Ikeda, Yasushi Makino, Hiroshi Matsubara

**Affiliations:** 1 Department of Chemistry, Graduate School of Science, Osaka Prefecture University, Sakai, Japan; 2 Department of Chemistry, Graduate School of Science, Osaka Metropolitan University, Gakuen-cho 1-1, Naka-ku, Sakai, Osaka 599-8531 Japan

**Keywords:** Chemical probing, Glycogen phosphorylase, Intersubunit communication, Phospho–dephospho hybrid, Phosphorylation regulation, Substrate recognition

## Abstract

Glycogen phosphorylase (GP) is biologically active as a dimer of identical subunits, each activated by phosphorylation of the serine-14 residue. GP exists in three interconvertible forms, namely GP*a* (di-phosphorylated form), GP*ab* (mono-phosphorylated form), and GP*b* (non-phosphorylated form); however, information on GP*ab* remains scarce. Given the prevailing view that the two GP subunits collaboratively determine their catalytic characteristics, it is essential to conduct GP*ab* characterization to gain a comprehensive understanding of glycogenolysis regulation. Thus, in the present study, we prepared rabbit muscle GP*ab* from GP*b*, using phosphorylase kinase as the catalyst, and identified it using a nonradioactive phosphate-affinity gel electrophoresis method. Compared with the half-half GP*a*/GP*b* mixture, the as-prepared GP*ab* showed a unique AMP-binding affinity. To further investigate the intersubunit communication in GP, its catalytic site was probed using pyridylaminated-maltohexaose (a maltooligosaccharide-based substrate comprising the essential dextrin structure for GP; abbreviated as PA-0) and a series of specifically modified PA-0 derivatives (substrate analogs lacking part of the essential dextrin structure). By comparing the initial reaction rates toward the PA-0 derivative (*V*_derivative_) and PA-0 (*V*_PA-0_), we demonstrated that the *V*_derivative_/*V*_PA-0_ ratio for GP*ab* was significantly different from that for the half-half GP*a*/GP*b* mixture. This result indicates that the interaction between the two GP subunits significantly influences substrate recognition at the catalytic sites, thereby providing GP*ab* its unique substrate recognition profile.

## Introduction

Glycogen, a highly branched polymer of d-glucose (G), serves as a form of energy storage in animals, fungi, and bacteria (Roach et al. 2012; Prats et al. 2018; Katz 2022). A key enzyme for utilizing glycogen is glycogen phosphorylase (GP; EC 2.4.1.1; MW 1.9 × 10^5^), which catalyzes the sequential phosphorolysis of glycogen to release α-d-glucose 1-phosphate (G-1-P) (Titani et al. 1977; Tagaya and Fukui 1984; Roach et al. 2012; Prats et al. 2018; Katz 2022). GP is biologically active as a dimer of identical subunits (Dombrádi 1981), and each subunit is activated by phosphorylase kinase (PhK; EC 2.7.11.19) through phosphorylation of the serine-14 (Ser^14^) residue (Krebs et al. 1964; Dombrádi 1981; Chan and Graves 1982; Nadeau et al. 2018). There are thus three isolatable GP forms, namely GP*a* (di-phosphorylated form, high activity), GP*ab* (mono-phosphorylated form, moderate activity), and GP*b* (non-phosphorylated form, low activity) (Dombrádi 1981). Muscle GP activity is regulated by interconversion between these three forms and the binding of various allosteric effectors, such as AMP, ATP, and d-glucose 6-phosphate (G-6-P) (Madsen et al. 1983). The most highly activated forms of muscle GP are AMP bound, regardless of the phosphorylation state (Lowry et al. 1964; Rush and Spriet 2001).

**Fig. 1:**
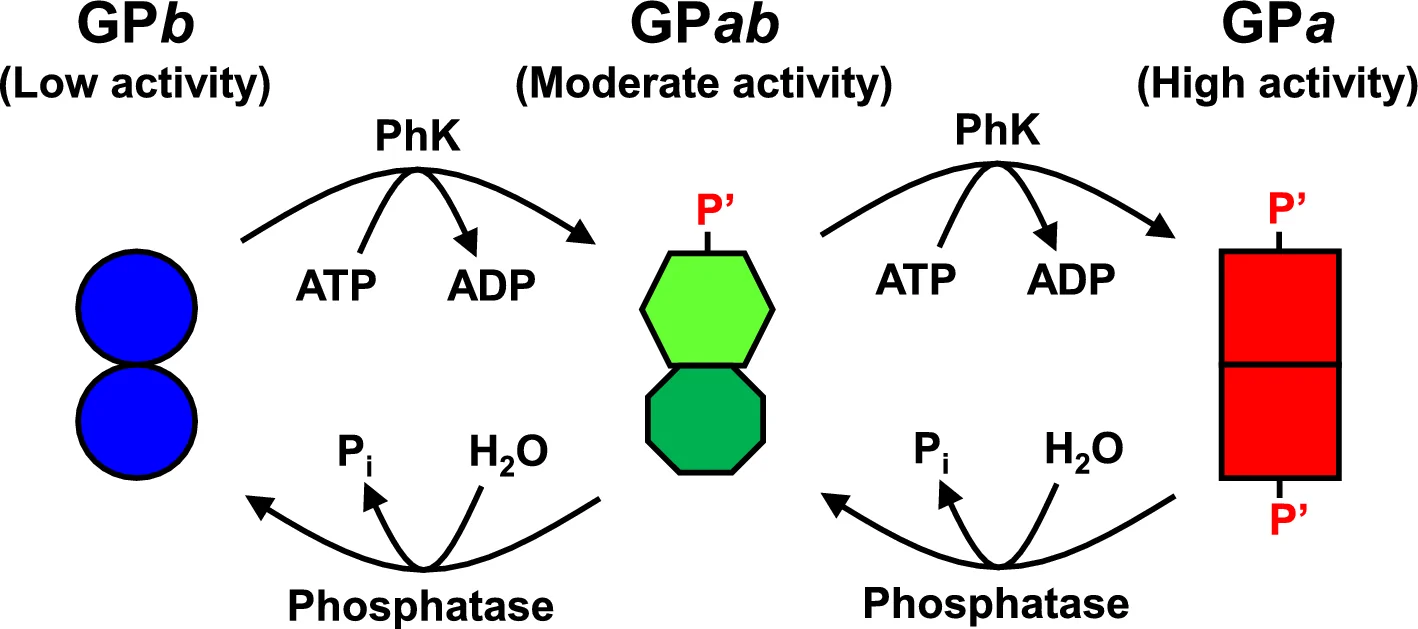
Prevailing view about phosphorylation regulation of GP. The two GP subunits collaboratively determine their catalytic characteristics, and therefore, the catalytic characteristics of GP*ab* (mono-phosphorylated form) are essentially different from those of GP*a* (di-phosphorylated form) and GP*b* (non-phosphorylated form) (Burkhardt and Wegener 1994)

GP*a* and GP*b* have been well characterized by structural (Madsen et al. 1983) and kinetic (Lowry et al. 1964; Madsen et al. 1983; Rush and Spriet 2001) approaches, including X-ray crystallography (Sprang et al. 1988, 1991; Barford et al. 1991). In contrast, little research progress has been achieved on GP*ab*, because the preparation and identification of the phospho–dephospho hybrid is highly challenging. Nevertheless, there is a prevailing view that the two GP subunits collaboratively determine their catalytic characteristics (Fig. 1), as supported by numerous experimental results (Burkhardt and Wegener 1994; Buchbinder et al. 1995; Rath et al. 2000; Mathieu et al. 2017; Kish et al. 2023). Therefore, GP*ab* characterization is indispensable for a comprehensive understanding of glycogenolysis regulation. Notably, Burkhardt and Wegener (1994) reported that the AMP-binding affinity of hawk moth muscle GP*ab* (*K*_d_, ~ 30 μM) was significantly different from those of both GP*a* (*K*_d_, ~ 0.3 μM) and GP*b* (*K*_d_, ~ 300 μM), suggesting that phosphorylation of either subunit of the GP dimer partially, but not fully, changed the AMP-binding site structures of both subunits. Accordingly, we are interested in whether the intersubunit communication in GP similarly influences substrate recognition at the catalytic sites, thereby providing GP*ab* with a unique substrate recognition profile.

GP assay is usually performed using macromolecular glycogen (MW 10^6^–10^7^) as the substrate. Notably, here, each GP subunit has two distinct maltooligosaccharide binding sites, namely a storage and catalytic site (Barford et al. 1991). Because the affinity for maltooligosaccharide is approximately 20 times higher in the storage site (*K*_d_, ~ 1 mM) than in the catalytic site (*K*_d_, ~ 20 mM), the GP dimer binds macromolecular glycogen mainly through the two storage sites to form a GP–glycogen complex (Makino et al. 2015). Conformational changes in one or both GP subunits may affect the positional relationship between the two storage sites, which might result in an affinity change of GP for macromolecular glycogen. However, quantitative evaluation of this effect on the GP activity is very difficult. Therefore, to focus on the catalytic site activity, small maltooligosaccharides comprising the minimum essential dextrin structure for GP should be used as the assay substrate (Makino et al. 2015). From this viewpoint, we previously developed pyridylaminated-maltohexaose (G-G-G-G-G-F, F = 1-deoxy-1-[(2-pyridyl)amino]-d-glucitol]; abbreviated as PA-0) and a series of specifically modified PA-0 derivatives (G_*m*_-Z-G_*n*_-F, *m* + *n* = 4 and Z = 3-acetoamido-3-deoxy-d-altrose) to investigate substrate recognition at the GP catalytic site (Nakamura et al. 2017). In the present study, by using PA-0 and its derivatives, the substrate recognition profile of GP*ab* was compared with those of GP*b*, GP*a*, and a half-half GP*a*/GP*b* mixture. In addition, their substrate recognitions were compared by using branched dextrins such as G-G-G-(G↔)G-G-G-F and G-G-G-G-(G↔)G-G-F (the double-headed arrow represents an α-1,6-glycosidic bond).

## Materials and methods

### Materials

Rabbit muscle glycogen phosphorylase *a* (GP*a*), glycogen phosphorylase *b* (GP*b*), phosphorylase kinase (PhK), AMP, ADP, and ATP were purchased from Sigma-Aldrich (St. Louis, MO, USA). The TSKgel DEAE-5PW column (7.5 × 75 mm) was acquired from Tosoh (Tokyo, Japan) and the Shodex NH2P-50 column (4.6 × 150 mm) was purchased from Showa Denko (Tokyo, Japan). The Vivaspin 6 centrifugal concentrator [3.0 × 10^4^ molecular weight cut-off (MWCO)] was acquired from Sartorius Stedim Lab (Gloucestershire, UK). Sodium dodecyl sulfate (SDS), WIDE-VIEW prestained protein size marker III, SuperSep Ace 7.5% polyacrylamide gel, and SuperSep Phos-tag (50 μmol/L) 6% polyacrylamide gel were purchased from FUJIFILM Wako Pure Chemical (Osaka, Japan). Coomassie brilliant blue (CBB; trade name EzStainAQua) was acquired from ATTO (Tokyo, Japan).

GP*a* and GP*b* were further purified according to previously described methods (Burkhardt and Wegener 1994). The fluorogenic oligosaccharides G_*n*_-F (*n* = 0–5) and G_*m*_-Z-G_*n*_-F (*m* + *n* = 4) were prepared using our previously reported methods (Nakamura et al. 2017). The fluorogenic branched dextrins G-G-(G↔)G-G-G-G-F (BD-3), G-G-G-(G↔)G-G-G-F (BD-4), G-G-G-G-(G↔)G-G-F (BD-5), and G-G-G-G-G-(G↔)G-F (BD-6) were also prepared using our previously reported methods (Yamamoto et al. 2007).

### Preparation of rabbit muscle GP*ab*

A mixture (300 μL) containing 50 mM Tris–HCl buffer (pH 8.2), 3.0 mM ATP, 10 mM MgCl_2_, 0.30 mM CaCl_2_, 40 mM sodium fluoride, 3.0 mM 2-mercaptoethanol, 0.30 mg rabbit muscle GP*b*, and 2.0 U rabbit muscle PhK was incubated at 37 °C for 10 min (Miyagawa et al. 2016). One unit of PhK was defined as the amount of enzyme that produces 1.0 μg of GP*a* per minute under the employed conditions. Next, the product solution was immediately subjected to anion-exchange HPLC using a TSKgel DEAE-5PW column (7.5 × 75 mm) at 25 °C and a flow rate of 0.9 mL/min. Two eluents, A and B, were employed. Eluent A comprised 5.0 mM sodium 2-glycerophosphate buffer, pH 6.8, containing 150 mM d-glucose and Eluent B comprised 30 mM sodium 2-glycerophosphate buffer, pH 6.8, containing 150 mM d-glucose and 500 mM NaCl. The column was first equilibrated with Eluent A. After injecting the sample, linear gradient elutions were performed using the following Eluent A:Eluent B proportions (v/v) and times: 100:0 in 5 min, 50:50 in 35 min, 0:100 in 5 min, 0:100 in 5 min, and 100:0 in 1 min. Each elution was monitored by measuring the absorbance at 280 nm. An intermediate protein exhibiting significant GP activity was collected as Fraction X. This fraction was then concentrated to 300 μL using a Vivaspin 6 centrifugal concentrator (3.0 × 10^4^ MWCO) and washed five times with a 3.0 mL solution containing 40 mM sodium phosphate buffer (pH 6.8) and 1.0 mM 2-mercaptoethanol. The enzyme solution was stored at 4 °C and used within 48 h.

### Polyacrylamide gel electrophoresis (PAGE)

Normal SDS-PAGE was performed after reduction of the sample with 2-mercaptoethanol, using a SuperSep Ace 7.5% polyacrylamide gel, as described by Laemmli (1970). WIDE-VIEW prestained protein size marker III was used as the MW marker. After electrophoresis, proteins in the gel were stained with CBB using EzStainAQua.

Improved Phos-tag SDS-PAGE (Zn^2+^–Phos-tag SDS-PAGE) was performed after reduction of the sample with 2-mercaptoethanol, using a SuperSep Phos-tag (50 μmol/L) 6% polyacrylamide gel (FUJIFILM Wako Pure Chemical), as described by Kinoshita and Kinoshita-Kikuta (2011). Following electrophoresis, the proteins in the gel were stained with CBB using EzStainAQua.

### GP assay in the direction of glycogenolysis

GP assay was performed in the direction of glycogenolysis according to our previously reported method (Nakamura et al. 2017). Briefly, a mixture (70 μL) containing 40 mM sodium phosphate buffer (pH 6.8), 35 μM PA-oligosaccharide, 1.0 mM 2-mercaptoethanol, 0.05% gelatin, 0–10 mM AMP, and 5–500 nM GP (i.e., 0.01–1 μM GP subunit) was incubated at 37 °C for 30 min. To terminate the reaction, the mixture was heated at 100 °C for 5 min. The chain-shortened product was then isolated and quantified by size-fractionation HPLC using a Shodex NH2P-50 (4.6 × 150 mm) column at a flow rate of 0.9 mL/min and 25 °C (Nakamura et al. 2017). The eluent, comprising acetonitrile:water:acetic acid (750:250:3, v/v/v), was titrated to pH 7.0 with 5.0% aqueous ammonia. PA-oligosaccharides were detected by fluorescence (excitation wavelength, 320 nm; emission wavelength, 400 nm). Three independent experiments were performed for each condition.

Although the fluorogenic substrate concentration was very low, it was considered to be constant throughout the reaction period because the chain-shortened product could be measured even at 10 fmol and > 95% of the original substrate remained unchanged at the end of the reaction period.

## Results and discussion

### Schematic representation of the GP catalytic site

**Fig. 2:**
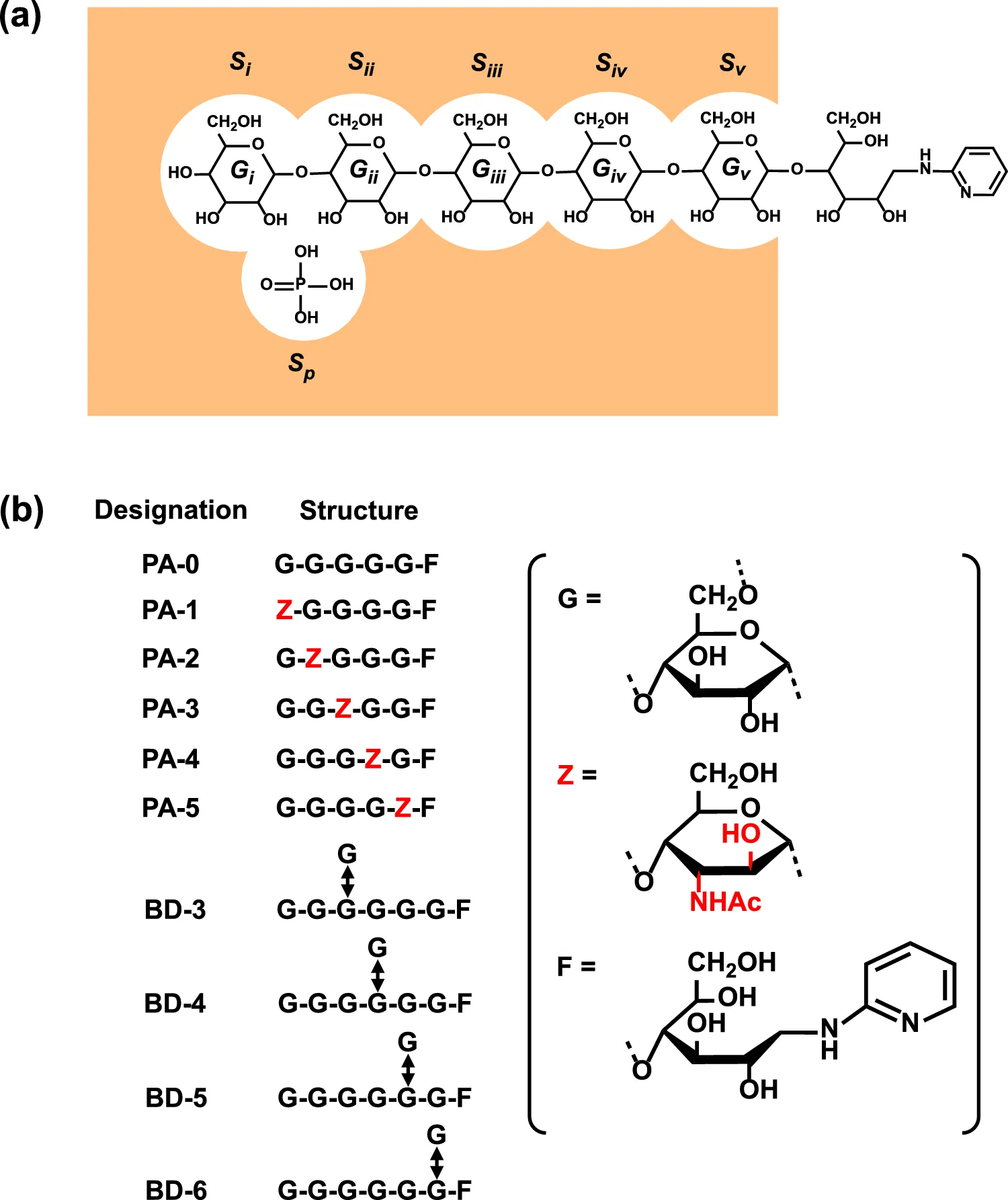
Probing of the GP catalytic site using fluorogenic oligosaccharides. **a** Schematic representation of the productive binding of PA-0 and P_i_ to the GP catalytic site. The GP catalytic site is composed of six subsites (*S*_*i*_*, S*_*ii*_*, S*_*iii*_*, S*_*iv*_*, S*_*v*_*,* and *S*_*p*_), wherein *S*_*i*_ and *S*_*p*_ are the sites for the nonreducing end G residue and P_i_, respectively (Weber et al. 1978; Johnson et al. 1988; Nakamura et al. 2017). When *G*_*i*_*, G*_*ii*_*, G*_*iii*_*, G*_*iv*_, and *G*_*v*_ of the maltooligosaccharide substrate and P_i_ interact with *S*_*i*_*, S*_*ii*_*, S*_*iii*_*, S*_*iv*_*, S*_*v*_, and *S*_*p*_, respectively, the α-1,4-glycosidic linkage between *G*_*i*_ and *G*_*ii*_ is phosphorolyzed. **b** Structures and designations of the fluorogenic oligosaccharides used in this study. G = d-glucose residue; Z = 3-acetoamido-3-deoxy-d-altrose residue; F = 1-deoxy-1-[(2-pyridyl)amino]-d-glucitol residue; hyphens represent α-1,4-glycosidic bonds; and the double-headed arrow represents an α-1,6-glycosidic bond

A schematic representation of the GP catalytic site is shown in Fig. 2a. The GP catalytic site is composed of six subsites (*S*_*i*_*, S*_*ii*_*, S*_*iii*_*, S*_*iv*_*, S*_*v*_, and *S*_*p*_) that are complementary to five tandem d-glucose residues (*G*_*i*_*, G*_*ii*_*, G*_*iii*_*, G*_*iv*_, and *G*_*v*_) and one phosphate (P_i_) molecule, respectively (Weber et al. 1978; Johnson et al. 1988; Nakamura et al. 2017). In the direction of glycogenolysis, the α-1,4-glycosidic linkage between *G*_*i*_ and *G*_*ii*_ is split, forming G-1-P. Although the catalytic action of GP is essentially reversible, a high concentration of P_i_ in the body inhibits GP activity in the direction of glycogen synthesis in animals. Previously, we reported that the apparent *K*_m_ and *V*_max_ values toward PA-0 were similar to those toward methyl α-maltopentaoside (G_5_-OCH_3_) (Nakamura et al. 2017). This indicates that the nonreducing end maltopentaosyl (G_5_-) residue of PA-0 suitably fits all the *S*_*i*_–*S*_*v*_ subsites.

### Preparation and identification of rabbit muscle GP*ab*

GP was the first enzyme studied in detail in relation to phosphorylation regulation (Krebs et al. 1964), with many GP studies performed using rabbit muscle GP*a* and/or GP*b* (Lowry et al. 1964; Madsen et.al. 1983; Sprang et al. 1988, 1991; Barford et al. 1991; Rush and Spriet 2001). Notably, while rabbit muscle GP*a*, GP*b*, and PhK are commercially available, GP*ab* is not. This is one of the major reasons for the stagnation of GP*ab* research. Previously, some dedicated researchers prepared GP*ab *in vitro by the partial phosphorylation of GP*b* using PhK as the catalyst (Vereb et al. 1987, 1992; Harris and Graves 1990). Notably, in the PhK-catalyzed GP*b* phosphorylation reaction, production of GP*a* (di-phosphorylated form, secondary product) is inevitable even at an early stage of the reaction, because the phosphorylation rate toward GP*ab* (mono-phosphorylated form, primary product) is faster than that toward GP*b* (non-phosphorylated form, original substrate) (Harris and Graves 1990). In addition, to correctly perform spectrophotometric protein detection (absorbance at 280 nm, A280), GP proteins should be completely separated from ATP and ADP, which have strong UV light-absorbing properties and are abundant in the product solution.

**Fig. 3:**
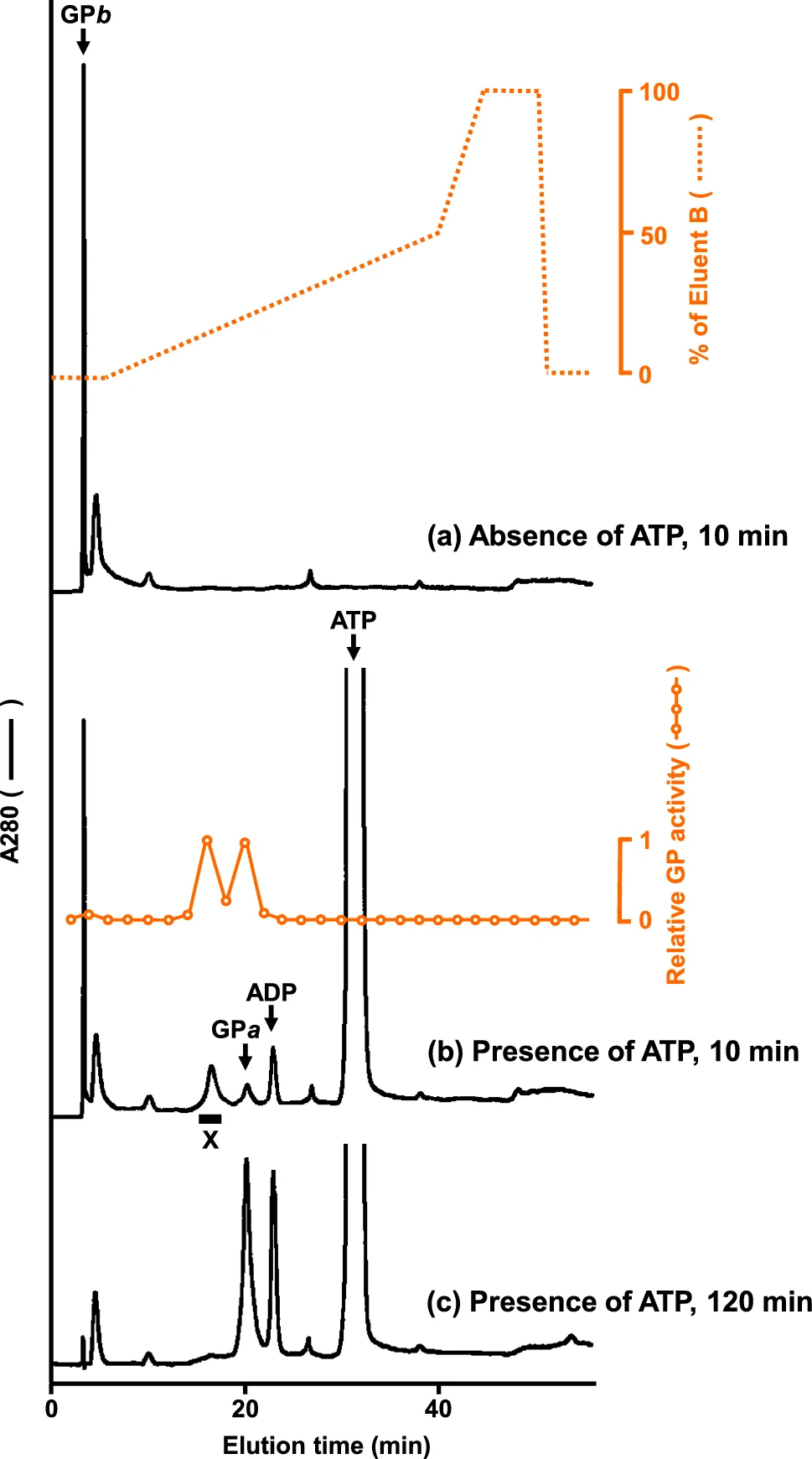
DEAE-5PW anion-exchange high-performance liquid chromatogram of the partial phosphorylation product of GP*b*. Rabbit muscle GP*b* was partially phosphorylated in vitro by rabbit muscle PhK, and the reaction product was immediately subjected to DEAE-5PW anion-exchange HPLC. **a** Product from 10 min reaction in the absence of ATP; dotted lines represent the gradient elution pattern. The small peak at 5 min was attributed to the presence of a contaminant. **b** Product from 10 min reaction in the presence of 3 mM ATP. Open circles indicate the relative GP activity in the eluted solution. The fraction indicated by the black bar was collected as Fraction X. **c** Product from 120 min reaction in the presence of 3 mM ATP

To perform the GP*ab* study correctly and consistently, we modified previously reported procedures for the preparation and identification of GP*ab*: First, PhK-catalyzed phosphorylation of rabbit muscle GP*b* was performed according to our previously reported method (Miyagawa et al. 2016), except that gelatin (protein adsorption-preventing agent) and ethylenediaminetetraacetic acid (EDTA; PhK inactivator) were not added to prevent disruption of the subsequent HPLC purification. Instead, the reaction product was immediately subjected to DEAE-5PW anion-exchange HPLC. The HPLC conditions were similar to those previously reported by Harris and Graves (1990), except that the initial concentration of sodium 2-glycerophosphate buffer was reduced from 30 to 5 mM; however, its concentration gradually increased (5–17.5 mM) in parallel with the NaCl gradient elution (0–250 mM). Notably, glycerophosphate ions in the mobile phase competitively inhibited four phosphorous components (i.e., GP*ab*, GP*a*, ADP, and ATP) to bind to the DEAE-5PW resins. Through our modifications, these four phosphorous components were separated from each other (Fig. 3), whereas separation and detection of ADP was not considered in the previous study (Harris and Graves 1990). An intermediate protein exhibiting significant GP activity was collected as Fraction X. Although some previous studies adopted an instantaneous increase in the eluent glycerophosphate concentration [e.g., jumping from 30 to 200 mM (Vereb et al. 1987, 1992)] to eluate GP*ab* from the anion-exchange column, we found that this practice caused co-elution of GP*ab* and GP*a* (data not shown).

**Fig. 4:**
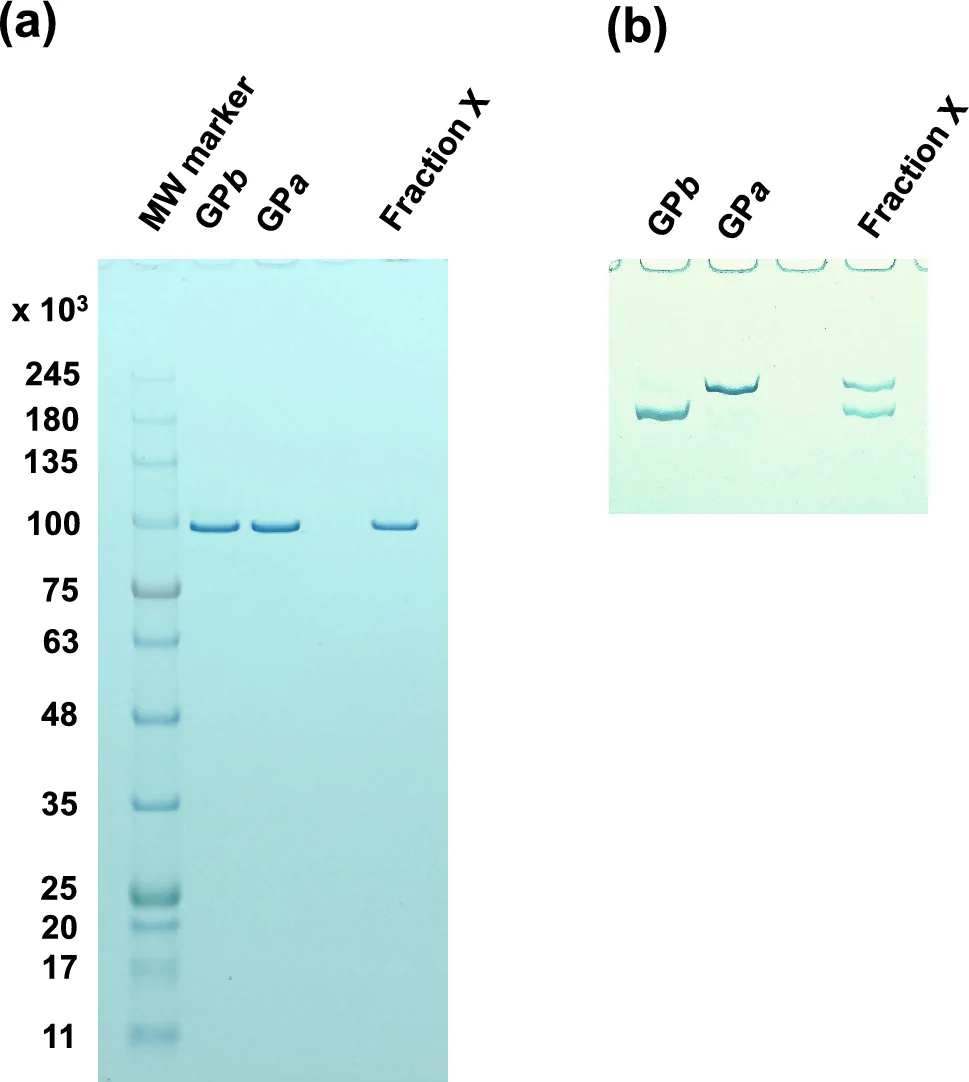
Identification of Fraction X by normal and Phos-tag SDS-PAGEs. GP*a*, GP*b*, and Fraction X were also analyzed using normal and Phos-tag SDS-PAGEs. The proteins in the gel were stained with CBB. **a** Normal SDS-PAGE: Fraction X migrated as a single band of MW 9.7 × 10^4^, which is concordant with the MW of a GP subunit. **b** Phos-tag SDS-PAGE: Phos-tag residues covalently linked to polyacrylamide gel interfered with the migration of the phosphorylated protein, resulting in the separation of the phosphorylated form, as a retarded band, from the non-phosphorylated form (Kinoshita and Kinoshita-Kikuta 2011). Fraction X split into two bands corresponding to the phosphorylated and non-phosphorylated GP subunits

GP*ab* has been usually identified through a radioactive labeling method using [γ-^32^P]ATP as the substate (Vereb et al. 1987, 1992; Harris and Graves 1990). However, the use of radioactive materials significantly hinders routine performance in GP*ab* research because researchers must follow strict rules to order, store, use, and dispose of these materials. To avoid this, in the present study, GP*ab* was identified by a new nonradioactive method using normal (Phos-tag-free) and Phos-tag SDS-PAGEs (Kinoshita and Kinoshita-Kikuta 2011). Phos-tag, a dinuclear metal complex of 1,3-bis[bis(pyridin-2-ylmethyl)amino]propan-2-olato, is a phosphate-binding molecule, and has been applied to nonradioactive analyses of protein phosphorylation reactions (Kinoshita et al. 2006). In Phos-tag SDS-PAGE, Phos-tag residues covalently linked to polyacrylamide gel interfere with phosphorylated protein migration, resulting in the separation of the phosphorylated form, as a retarded band, from the non-phosphorylated form. The results of both SDS-PAGEs are shown in Fig. 4. For the normal SDS-PAGE, Fraction X migrated as a single band of MW 9.7 × 10^4^, which was concordant with the MW of a GP subunit. However, with Phos-tag SDS-PAGE, Fraction X split into two bands corresponding to the phosphorylated and non-phosphorylated GP subunits. From these results, Fraction X was identified as the phospho–dephospho hybrid of GP, specifically, GP*ab*.

### AMP-induced activation of rabbit muscle GP*ab*

**Fig. 5:**
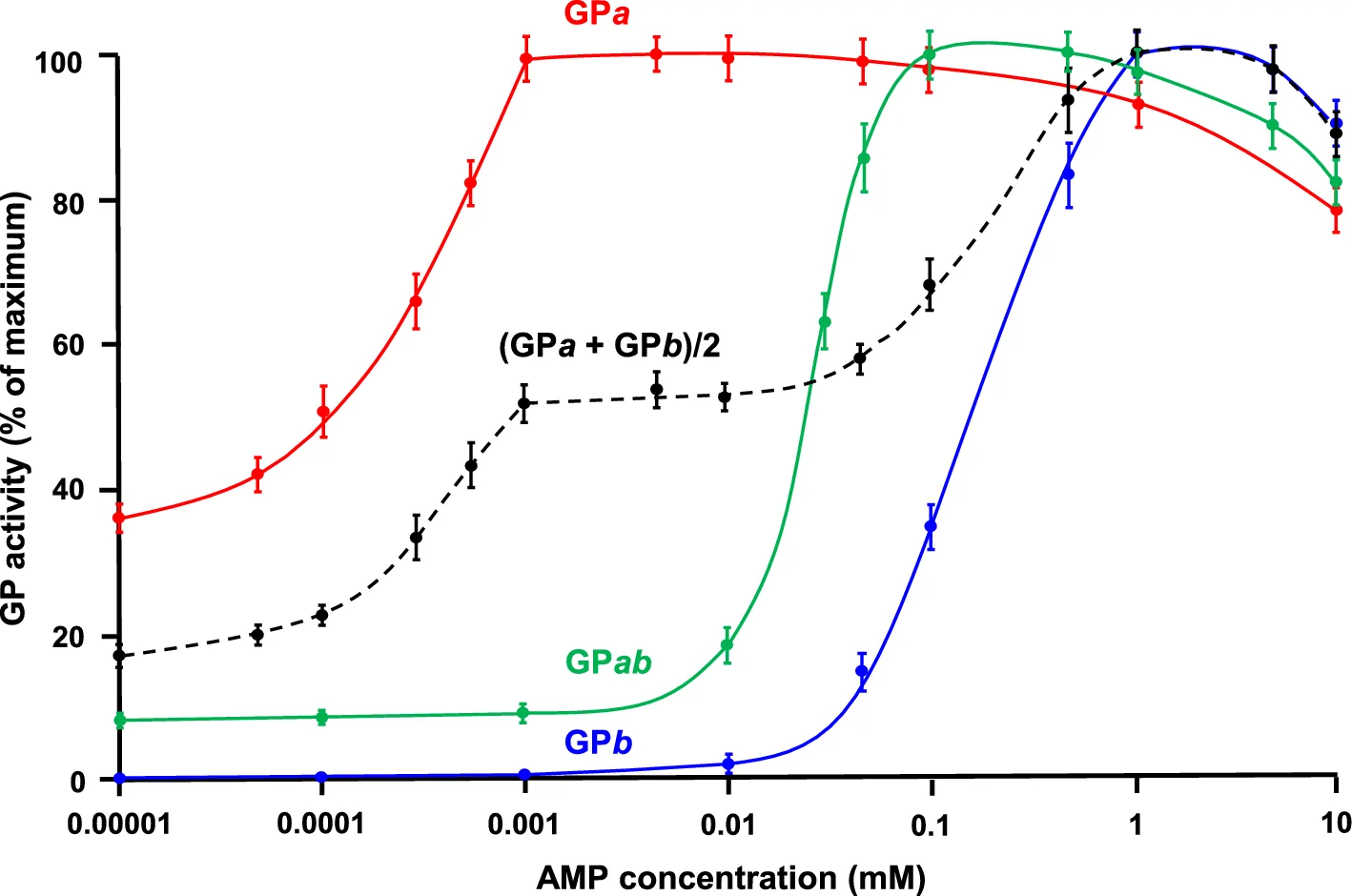
Effect of AMP on the catalytic activity of rabbit muscle GPs with various phosphorylation states. The initial reaction rates toward PA-0 at various AMP concentrations for GP*a*, GP*b*, and GP*ab* were compared. Each series is expressed as a percentage of the respective maximum activity. A common logarithmic scale is used for the abscissa to show a very wide ATP concentration range. Each value shows the mean ± SD (*n* = 3)

Muscle GP is allosterically activated by AMP (Lowry et al. 1964; Madsen et al. 1983; Rush and Spriet 2001). Notably, muscle GP*a* exhibits a very strong affinity for AMP (*K*_d_, ~ 0.3 μM) compared with muscle GP*b* (*K*_d_, ~ 300 μM) (Miyagawa et al. 2016). As previously stated, Burkhardt and Wegener (1994) reported that GP*ab* from hawk moth muscle showed a single and unique AMP dependence (*K*_d_, ~ 30 μM), suggesting that phosphorylation of either subunit of the GP dimer partially, but not fully, changed the AMP-binding site structures of both subunits. As for rabbit muscle GP*ab*, the AMP dependence was previously compared with that of GP*b* (Vereb et al. 1987); however, comparison of the three phosphorylation forms (i.e., GP*a*, GP*ab*, and GP*b*) is necessary to fully characterize GP*ab*. In addition, comparison of GP*ab* with a half-half GP*a*/GP*b* mixture is expected to provide clear criteria for assessing the interaction between the phosphorylated and non-phosphorylated subunits (Fig. 1). Hence, in the present study, we compared the AMP dependence in rabbit muscle GP*a*, GP*ab*, GP*b,* and a half-half GP*a*/GP*b* mixture. To focus on the catalytic site activity, PA-0, comprising the minimum essential dextrin structure for GP, was used as the assay substrate (Fig. 2); in contrast, macromolecular glycogen was used in the previous GP*ab* studies (Vereb et al. 1987, 1992; Harris and Graves 1990; Burkhardt and Wegener 1994). The results are summarized in Fig. 5: The AMP-induced activation profile of rabbit muscle GP*ab* showed a single-step rise curve, which fell between those of rabbit muscle GP*a* and GP*b*. This agrees with the results of the study by Burkhardt and Wegener (1994) on insect muscle GP*ab* activity toward macromolecular glycogen. In addition, the AMP-induced activation profile of the half-half GP*a*/GP*b* mixture presented a two-step rise curve, which was remarkably different from that of GP*ab*. These results indicated that the two heterogenous subunits of rabbit muscle GP*ab* significantly interacted with each other (Fig. 1), inducing the unique AMP-binding affinity of both subunits.

### Substrate recognition at the GP*ab* catalytic sites

**Fig. 6:**
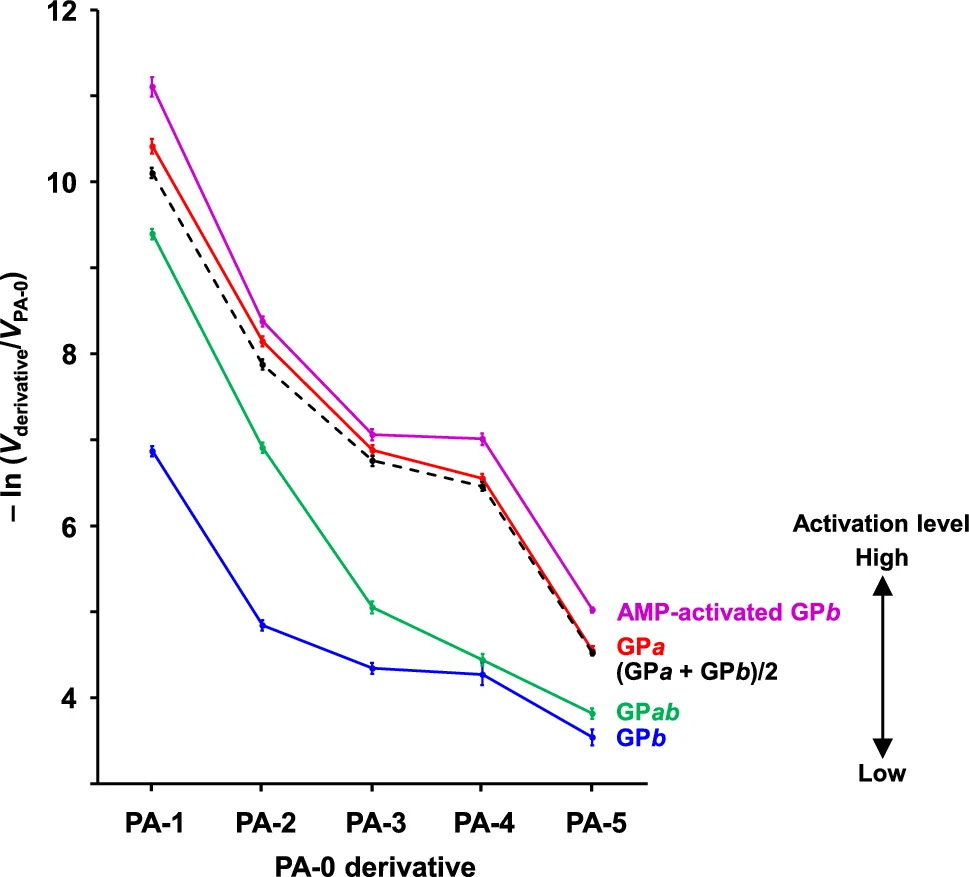
Effect of the AltNAc residue position on the GP activity. Using GPs at various activation levels, the initial reaction rates toward the PA-0 derivatives were compared with that toward PA-0. Chemical structures of PA-0 and its derivatives are shown in Fig. 2b. A natural logarithmic scale is used for the ordinate to show a wide range of the *V*_derivative_/*V*_PA-0_ ratios. Data for AMP-activated GP*b*, GP*a*, and GP*b* were reproduced from our previous report (Nakamura et al. 2017). Each value shows the mean ± SD (*n* = 3). Our previous study revealed that with increasing GP activation level, recognition of the substrate maltopentaosyl (G-G-G-G-G-) residue by the GP catalytic site became increasingly stricter

The maltooligosaccharide-binding region of the GP catalytic site accommodates and recognizes the nonreducing end maltopentaosyl (G_5_-) residue of a substrate, as shown in Fig. 2a (Nakamura et al. 2017). By comparing the initial reaction rate toward the PA-0 derivative (*V*_derivative_) with that toward PA-0 (*V*_PA-0_), we previously reported that *V*_derivative_/*V*_PA-0_ decreased with increasing GP activation level (Nakamura et al. 2017). Specifically, as the GP activation level increases, GP catalytic site recognition of the G_5_ residue must become stricter. In the present study, using PA-0 (G_5_-F) and its derivatives (G_*m*_-Z-G_*n*_-F, *m* + *n* = 4) as substrates, the substrate recognition profile of GP*ab* was compared with those of GP*a*, GP*b*, and the half-half GP*a*/GP*b* mixture. The *V*_derivative_/*V*_PA-0_ ratio for GP*ab* fell between the GP*a* and GP*b* ratios, as expected (Table 1 and Fig. 6). Remarkably, the *V*_derivative_/*V*_PA-0_ ratio for GP*ab* was much greater than that for the half-half GP*a*/GP*b* mixture, suggesting that the phosphorylated subunit of GP*ab* was not sufficiently activated compared with that of GP*a*. This suggestion was supported by the lower activity of GP*ab* compared to that of the half-half GP*a*/GP*b* mixture (Footnotes d and e in Table 1).

**Table 1:** Degradation of specifically modified dextrin by GP*ab* and the half-half GP*a*/GP*b* mixture

Substrate^a^	Relative initial reaction rate^b^	
	GP*ab*	(GP*a* + GP*b*)/2
PA-0 (G_5_-F)	1.00 ± 0.05^c,d^	1.00 ± 0.04^c,e^
PA-1	0.000083 ± 0.000005^f,g^	0.000041 ± 0.000002^f,g^
PA-2	0.00100 ± 0.00004^h^	0.00038 ± 0.00002^h^
PA-3	0.0064 ± 0.0004^i^	0.00116 ± 0.00006^i^
PA-4	0.0118 ± 0.0007^j^	0.00157 ± 0.00008^j^
PA-5	0.022 ± 0.001^k^	0.0109 ± 0.0003^k^

^a^Substrate structures are shown in Fig. 2b

^b^Values are the rates relative to the rate for PA-0 at 35 mM. Each value shows the mean ± SD (*n* = 3)

^c^No enzymatic activity was observed when P_i_ was omitted from the assay medium

^d^Initial reaction rate was 4.11 nmol G_4_-F/(min mg protein)

^e^Initial reaction rate was 9.29 nmol G_4_-F/(min mg protein)

^f^G_3_-F (secondary product) was also observed because G_4_-F (primary product) was phosphorolyzed much faster than PA-1

^g–k^Values followed by the same letter are significantly different (*p* < 0.05)

**Fig. 7:**
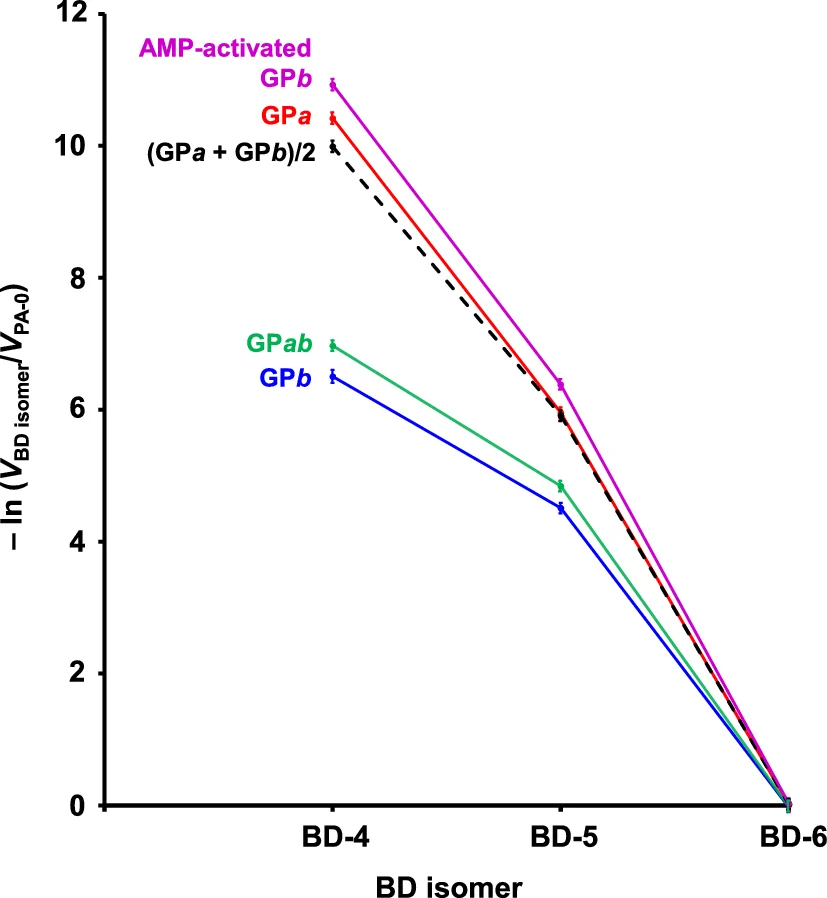
Effect of the α-1,6-linked G residue position on the GP activity. Using GPs at various activation levels, the initial reaction rates toward BD isomers were compared with that toward PA-0. Chemical structures of PA-0 and BD isomers are shown in Fig. 2b. A natural logarithmic scale is used for the ordinate to show a wide range of the *V*_BD isomer_/*V*_PA-0_ ratios. Each value shows the mean ± SD (*n* = 3)

We next proceeded to compare the substrate recognitions of GP*a*, GP*ab*, GP*b*, and the half-half GP*a*/GP*b* mixture using branched dextrins G-G-G-(G↔)G-G-G-F (BD-4), G-G-G-G-(G↔)G-G-F (BD-5), and G-G-G-G-G-(G↔)G-F (BD-6). The results are summarized in Table 2 and Fig. 7. The *V*_BD-6_/*V*_PA-0_ ratios approximated unity regardless of the GP activation level, indicating that the α-1,6-linked G residue of BD-6 was located outside of the GP catalytic site. Interestingly, the *V*_BD-5_/*V*_PA-0_ ratios were significantly lower than the *V*_PA-5_/*V*_PA-0_ ratios. This might be because the α-1,6-linked G residue is bulky, and thus, in addition to the *S*_*v*_–*G*_*v*_ interaction, the *S*_*iv*_–*G*_*iv*_ interaction might also be somewhat disturbed (Fig. 2a). Similar to the *V*_PA-4_/*V*_PA-0_ and *V*_PA-5_/*V*_PA-0_ ratios (Table 1 and Fig. 6), the *V*_BD-4_/*V*_PA-0_ and *V*_BD-5_/*V*_PA-0_ ratios for GP*ab* were significantly greater than those for the half-half GP*a*/GP*b* mixture (Table 2 and Fig. 7).

**Table 2:** Branched dextrin degradation by GPs at various activation levels

Substrate^a^	Relative initial reaction rate^b^				
	AMP-activated GP*b*^c^	GP*a*	GP*ab*	(GP*a* + GP*b*)/2	GP*b*
PA-0 (G_5_-GPA)	1.00 ± 0.04^d,e^	1.00 ± 0.04^d,f^	1.00 ± 0.05^d,g^	1.00 ± 0.04^d,h^	1.00 ± 0.04^d,i^
BD-3	Not evaluated^j^	Not evaluated^j^	Not evaluated^j^	Not evaluated^j^	Not evaluated^j^
BD-4	0.000018 ± 0.000001	0.000030 ± 0.000002	0.00094 ± 0.00005^k^	0.000046 ± 0.000003^k^	0.0015 ± 0.0001
BD-5	0.0017 ± 0.0001	0.0026 ± 0.0001	0.0079 ± 0.0004^l^	0.0027 ± 0.0001^l^	0.0112 ± 0.0005
BD-6	0.97 ± 0.06	0.99 ± 0.05	1.02 ± 0.04	0.98 ± 0.05	1.03 ± 0.03

^a^Substrate structures are shown in Fig. 2b

^b^Values are the rates relative to the rate for PA-0 at 35 mM. Each value shows the mean ± SD (*n* = 3)

^c^GP*b* activity was assayed in the presence of 1 mM AMP

^d^No enzymatic activity was observed when P_i_ was omitted from the assay medium

^e^Initial reaction rate was 50.2 nmol G_4_-F/(min mg protein)

^f^Initial reaction rate was 18.4 nmol G_4_-F/(min mg protein)

^g^Initial reaction rate was 4.11 nmol G_4_-F/(min mg protein)

^h^Initial reaction rate was 9.29 nmol G_4_-F/(min mg protein)

^i^Initial reaction rate was 203 pmol G_4_-F/(min mg protein)

^j^Production of G-(G↔)G-G-G-G-F was less than the detection limit (< 10 fmol)

^k,l^Values followed by the same letter are significantly different (*p* < 0.05)

As we expected, the three phosphorylation forms of GP displayed the respective substrate recognition profiles. Notably, substrate recognition of GP*ab* was significantly different from that of the half-half GP*a*/GP*b* mixture. These results indicated that the intersubunit communication in GP significantly influenced substrate recognition at the catalytic sites. A deeper structural investigation (e.g., via X-ray crystallography or hydrogen–deuterium-exchange mass spectrometry) would be helpful to obtain further information.

Notably, because GP catalyzes the first step in glycogenolysis, it has become a potential key target for treating type 2 diabetes (Oikonomakos and Somsák 2008). Hence, the search for potent inhibitors of the GP catalytic site is attracting particular attention. Based on the in vitro inhibitory effects toward GP*a* and AMP-activated GP*b*, several d-glucose analogs, such as β-d-glucopyranosylamines, *C*-β-d-glucopyranosyl derivatives, and iminosugars, were reported as hopeful candidates (Somsák 2011). These d-glucose analogs predominantly bind to the *S*_*i*_ subsite (Fig. 2a) and competitively inhibit a maltooligosaccharide substrate from binding to the catalytic site. In the present study, it was revealed that GP*ab* catalytic sites have unique catalytic characteristics due to the interaction between the phosphorylated and non-phosphorylated subunits. Accordingly, we are planning to investigate the inhibitory effects of these d-glucose analogs toward GP*ab*, which may provide important information toward their practical use in the medical field.

## Data Availability

The data that supported the findings of this study are available from the corresponding author upon reasonable request.
